# TEACH Kitchen: A Chronological Review of Accomplishments

**DOI:** 10.21633/jgpha.6.408

**Published:** 2017

**Authors:** Jung Hee Chae, Benjamin E. Ansa, Selina A. Smith

**Affiliations:** 1 Medical College of Georgia, Augusta University, Augusta, GA; 2 Institute of Public & Preventive Health, Augusta University, Augusta, GA; 3 Department of Family Medicine, Medical College of Georgia, Augusta, GA

**Keywords:** nutrition, cooking kitchen, chronic disease, obesity, hypertension, hyperlipidemia, diabetes mellitus

## Abstract

**Background:**

The Eating and Cooking Healthy (TEACH) Kitchen was founded at the Medical College of Georgia in 2015 as a nutrition-based intervention to combat the high prevalence of obesity and obesity-related chronic diseases in the area of Augusta, Georgia. Despite the importance of diet in the management of chronic diseases, inadequate nutrition education among patients and healthcare providers presents a barrier. The purpose of TEACH Kitchen is to address this gap.

**Methods:**

TEACH Kitchen is as a student-led initiative that promotes healthy cooking among medical students and patients with chronic diseases. Healthy nutrition and cooking classes are held during the academic year. Participants spend four weeks on each of four modules: obesity, hypertension, hyperlipidemia, and diabetes mellitus. Data collection, which began in January 2017, is currently on going. TEACH Kitchen has collaborated with Augusta University, Sodexo, and Kohl’s.

**Results:**

Currently, TEACH Kitchen has enrolled 14 patients and 6 children. Anticipated results include measurements of pre-and post-intervention changes in knowledge, attitudes, beliefs, and competence in nutrition, as well as differences in clinical indicators, including body mass index, blood pressure, lipid profile, and HbA1c.

**Conclusions:**

TEACH Kitchen is the first medical school-based nutrition/cooking education initiative in Augusta, Georgia. It provides patients and medical students with hands-on healthy nutrition/cooking experience with the goal of decreasing the prevalence and improving the outcome of obesity-related diseases.

## INTRODUCTION

The Eating and Cooking Healthy (TEACH) Kitchen at the Medical College of Georgia (MCG) at Augusta University was founded in 2015 to address the high prevalence of obesity and obesity-related chronic diseases in the Augusta area of Georgia (GA). GA ranks 19^th^ in the US in obesity rates, with 30.5% of adults and 12.7% of adolescents recorded as being obese in 2014 (Levi et al., 2015; CDC, 2014). More than 35% of adults are obese in Richmond County, GA (the site of TEACH Kitchen). This is higher than the national and state averages (CDC, 2015). The burden of obesity is a public health concern because of its associated risk for several chronic illnesses, including hypertension, cardiovascular and cerebrovascular diseases, diabetes mellitus, and some cancers (Malnick et al., 2006). GA ranks 12th in hypertension and 10th in diabetes (Levi et al., 2015). In 2013, 35.5% of Georgians had hypertension, and about 11% had diabetes mellitus (CDC, 2013; 2014). The healthcare costs associated with obesity-related diseases are also of concern. Generally, increased body mass index (BMI) correlates with increased healthcare costs, including medical claims and sick-day absences from employment. Obese adults spend 42% more on healthcare than adults at a healthy BMI (Levi et al., 2015).

Various interventions address obesity and obesity-related chronic diseases; however, a barrier faced by many people of lower socioeconomic status is lack of nutrition education (Murray et al., 2016). Traditional nutrition education typically involves medical nutrition therapy (MNT) led by a registered dietitian (RD). RD-led MNT programs have higher attrition rates (Meffert et al., 2010; Cui et al., 2015; Norris et al., 2002). Recent research has explored innovative approaches to nutrition education through hands-on food preparation, a more effective approach for affecting lifestyle changes (Murray et al., 2016; Raber et al., 2016; Curtis et al., 2012). For children, cooking education is expected to promote healthy food choices, which is relevant because adulthood obesity often begins as childhood obesity (Ratner et al., 2016; Levi et al., 2015; Murray et al., 2016; Hersch et al., 2014).

Despite the need for nutrition-based interventions, most medical school curricula do not include sufficient nutrition education (Jacob et al., 2016). Only two of five medical schools require the minimum 25 hours of nutrition education, which is a standard recommendation by the National Academy of Sciences (Monlezun et al., 2015). Only 22.2% of graduating medical students report readiness to offer adequate nutrition education to patients (Mogre et al., 2017). Furthermore, about 70% of surveyed medical students reported dissatisfaction with the amount of nutrition education received in medical school (Mogre et al., 2017). Hands-on nutrition education can bridge this gap between medical knowledge and clinical application (Jacob et al., 2016; Monlezun et al., 2015; Leong et al., 2014; Levine et al., 2015).

The Goldring Center for Culinary Medicine (GCCM) at Tulane University School of Medicine was the first medical school-based teaching kitchen for underserved communities. GCCM incorporates the teaching kitchen for patients, with a four-year longitudinal curriculum for medical students (Monlezun et al., 2015). Patients who participated in GCCM had reductions of HbA1c (p=0.575), diastolic blood pressure (p=0.037), and total cholesterol (p=0.044) compared to the control group, who participated in RD-led MNT (Monlezun et al., 2015). These results are promising advances in health outcomes. Medical students who participated in GCCM were more likely to report higher proficiency in counseling patients about nutrition (p=0.012), weight loss (p=0.021), and aerobic exercise (p=0.001) (Birkhead et al., 2014). Several other teaching kitchens have now been established (Polak et al., 2015).

Obesity and obesity-related chronic diseases are a public health concern in GA. Traditional nutrition education may not be an effective approach for translating nutrition principles to healthy lifestyle changes. In this context, TEACH Kitchen was founded as the first culinary medicine program at MCG. The purpose of this report is to present a chronological review of the development and implementation of TEACH Kitchen.

## METHODS

### Setting

The setting for TEACH Kitchen is the MCG at Augusta University in Augusta, GA. Cooking sessions are held at the Terrace Dining area, located on the second floor of the Augusta University Medical Center (AUMC).

### Community, participant characteristics, and recruitment

The community includes residents of Augusta, GA, and surrounding areas. Adult participants are patients diagnosed with obesity, diabetes mellitus, hyperlipidemia, or hypertension, and are receiving care at AUMC. Adolescent participants are the children of adult participants in the study. Patients are recruited to TEACH via referral from healthcare providers (primary care physicians in the departments of Family Medicine, Internal Medicine, and Cardiac Rehab). Patients are also referred from student-led clinics (Clinica Latina, Equality Clinic, 8^th^ Street Clinic, Asian Clinic, FaithCare, and Women’s Clinic) (White et al., 2016).

### Processes, interventions, and comparisons

TEACH investigators developed nutrition educational materials and planned recipes for the cooking sessions corresponding to obesity, diabetes mellitus, hyperlipidemia, or hypertension. The intervention is participation in TEACH cooking sessions. Each cooking session is structured in three parts. The session begins with nutrition education (20 minutes), in which participants learn about foods to include and exclude in their diet, and how to read nutrition labels. This is followed by the hands-on cooking session (60 minutes), in which participants learn food preparation techniques. Lastly, there is a guided post-cooking discussion (40 minutes), in which participants discuss principles such as portion size, healthy shopping techniques, and meal planning. Each session lasts 2 hours and is held weekly for 4 weeks. TEACH facilitators are presently analyzing pre- and post-intervention data. These data include clinical metrics such as HbA1c, lipid profile, blood pressure, weight and BMI. The data also include psychometric assessments of pre- and post-intervention attitudes and competence regarding healthy cooking.

### Timeline

**Figure 1:**
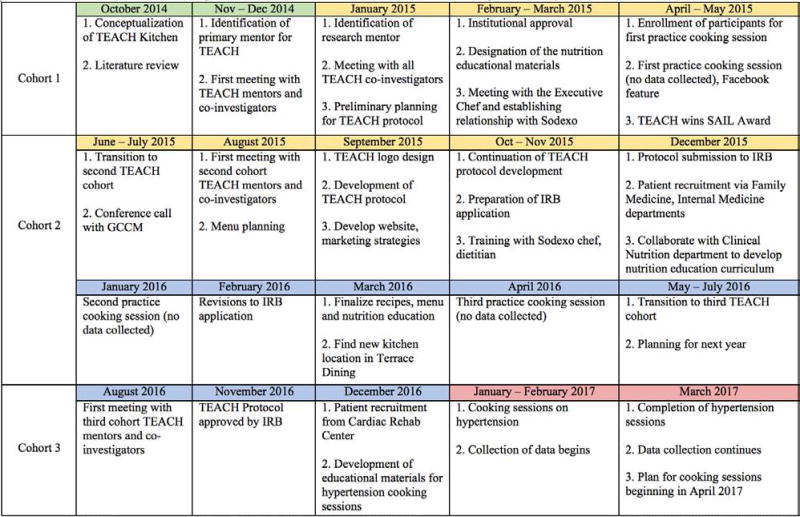
Timeline of TEACH Kitchen, organized by cohort

TEACH Kitchen started in October 2014, and, presently, patients from the Augusta University Health are being educated through cooking sessions about managing their chronic diseases through healthy eating. A chronological review of TEACH is presented below (Figure 1).

**Figure 2:**
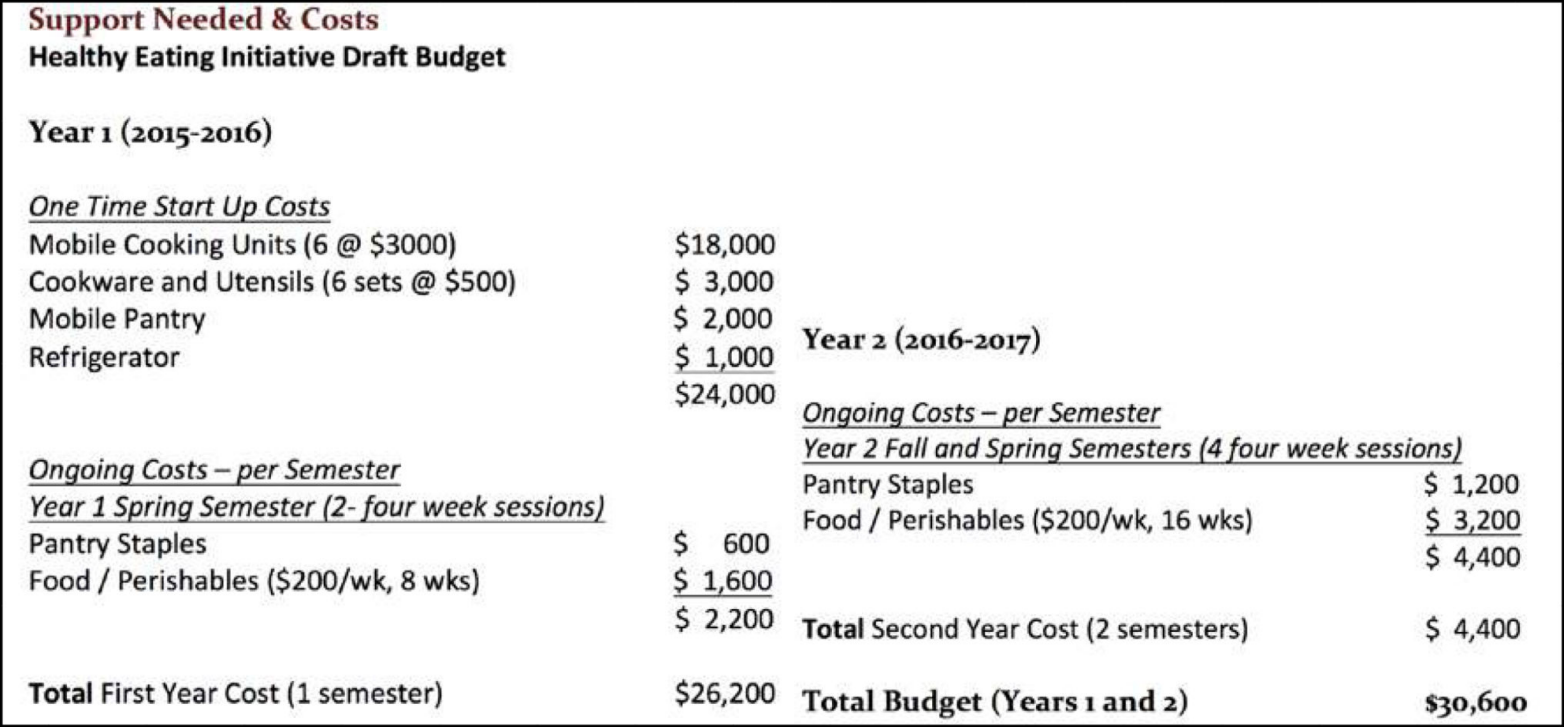
Estimated budget for the first 2 years

The inspiration for founding a hands-on teaching kitchen began with a seminar at the 2014 APAMSA National Conference entitled “Hey Doc, what should I eat? How to Talk About Food with Your Patients in a Clinical Setting,” by Dr. Ben Leong, who leads the cooking kitchen at GCCM. Preliminary meetings to pursue a cooking kitchen at MCG began in late 2014 with our faculty mentor, Dr. Selina Smith, PhD, MDiv, Director of the Institute of Public and Preventive Health (IPPH). We also started discussions about potential research questions with Dr. Benjamin Ansa, MD, a senior research associate at the IPPH. Initial meetings consisted of formulating the mission statement and consideration of logistics and estimated costs (Figure 2).

At MCG, TEACH was officially registered as a student organization at six months after conceptualization. During this time, TEACH coordinators created nutrition educational materials, planned for the first practice cooking session (pilot session), and established relationships with Sodexo, a company that manages the university’s food services. Considering the patient population in Augusta, GA, we focused the cooking sessions on four chronic diseases: obesity, hypertension, hyperlipidemia, and diabetes mellitus.

**Figure 3:**
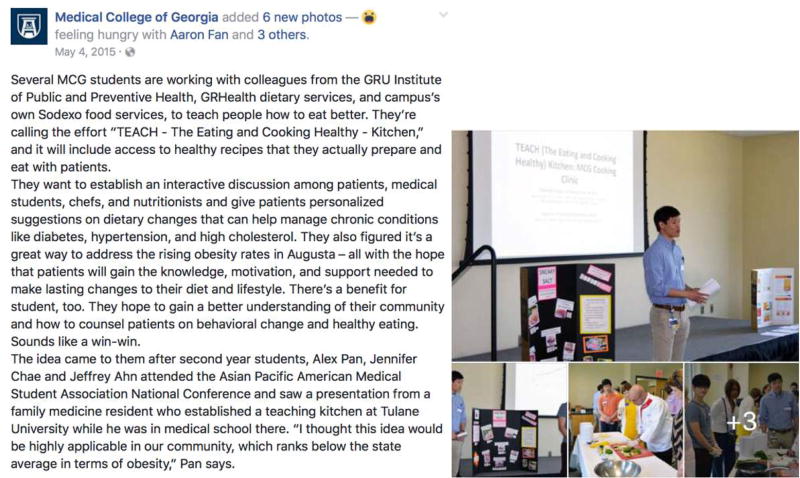
TEACH pilot study featured on MCG Facebook

For the pilot session, we planned to spend the first 15–20 minutes reviewing a “Hypertension nutrition therapy” handout, with tips on limiting sodium intake. After this educational component, we planned to begin cooking based on a menu planned by Sodexo executive chef David Moulton and dietitian Pam Brisky. The recipes included chicken fajitas with salad. Ingredients such as onion, tomato, and chicken were pre-sliced to ease the transition into the cooking component. As a first step in establishing a new relationship with patients in our community, the pilot session ran on April 21, 2015. The session was featured on the MCG Facebook page (Figure 3). Shortly afterwards, TEACH Kitchen was recognized by the university and awarded “Organization of the Year.”

During the first year, the coordinators comprised non-selected medical students who were interested in building TEACH. After the founding of TEACH, coordinators were selected based on an application and voting process. The application used to select the second cohort of coordinators consisted of four points:

1. Describe your previous leadership experience.
2. List any cooking experience that you have.
3. What experience do you have working with patients?
4. Why would you like to be a coordinator for TEACH Kitchen at MCG?

**Figure 4:**
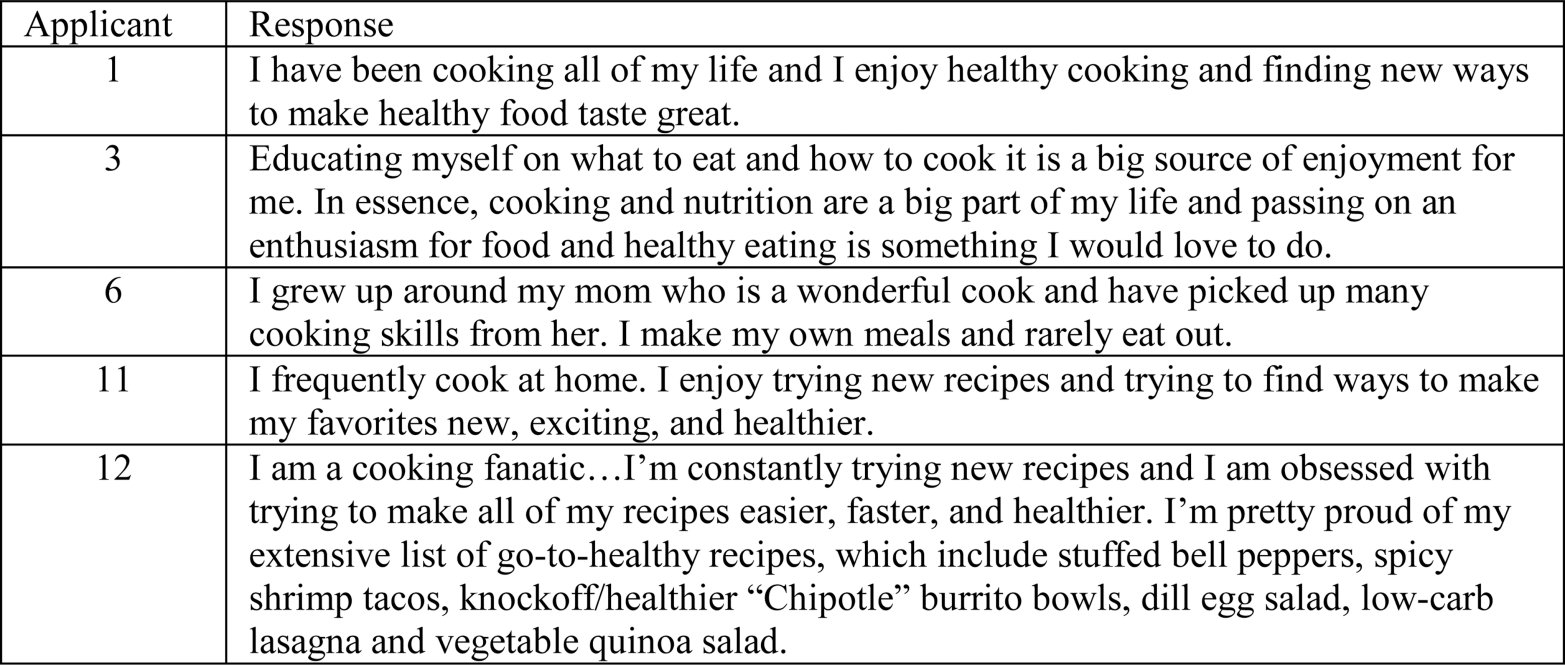
Selected applicant responses to “List any cooking experience that you have”

**Figure 5:**
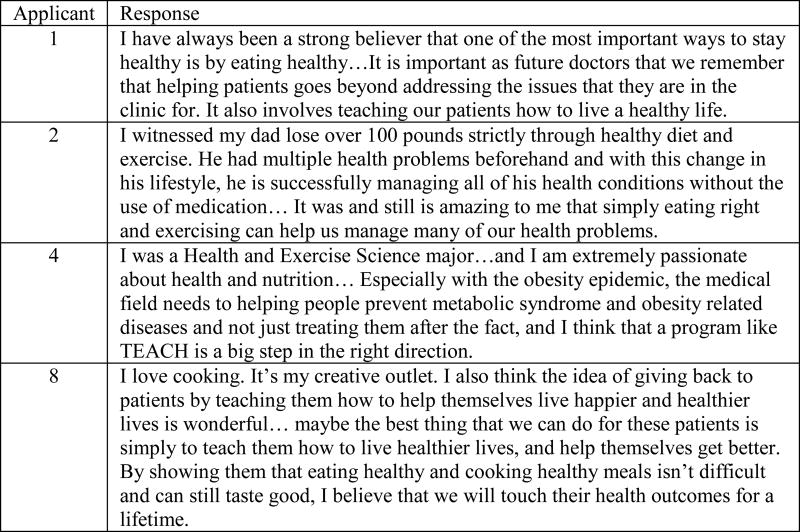
Selected applicant responses to “Why would you like to be a coordinator for TEACH Kitchen at MCG”

We were interested in selecting students with either cooking experience or a passion for changing lifestyles through healthy cooking and eating. All applicants had leadership experience from college or medical school, and all had a personal history of cooking experience. Many expressed a fondness for cooking various cuisines, or cooking with their family and friends. Figure 4 is a table of excerpted applicant responses to personal cooking experience. In regard to the question of why the applicants were applying for the position, a common theme was the desire to unite two interests: cooking and community service. Figure 5 presents applicant responses to interest in being a TEACH coordinator.

In July 2015, the second cohort of TEACH coordinators communicated with Dr. Timothy Harlan of GCCM on a conference call. In regard to funding, TEACH Kitchen established relationships with Sodexo, the IPPH, and Kohl’s Department Store. Funding from Kohl’s allows TEACH to incentivize and reward patient attendance of cooking sessions: a $25 gift card is provided to each adult participant upon completion of a 2-hour session, and children participants are presented Kohl’s Healthy Kids Kitchen products (t-shirt, chef’s hat, spatula, and measuring cup).

**Figure 6:**
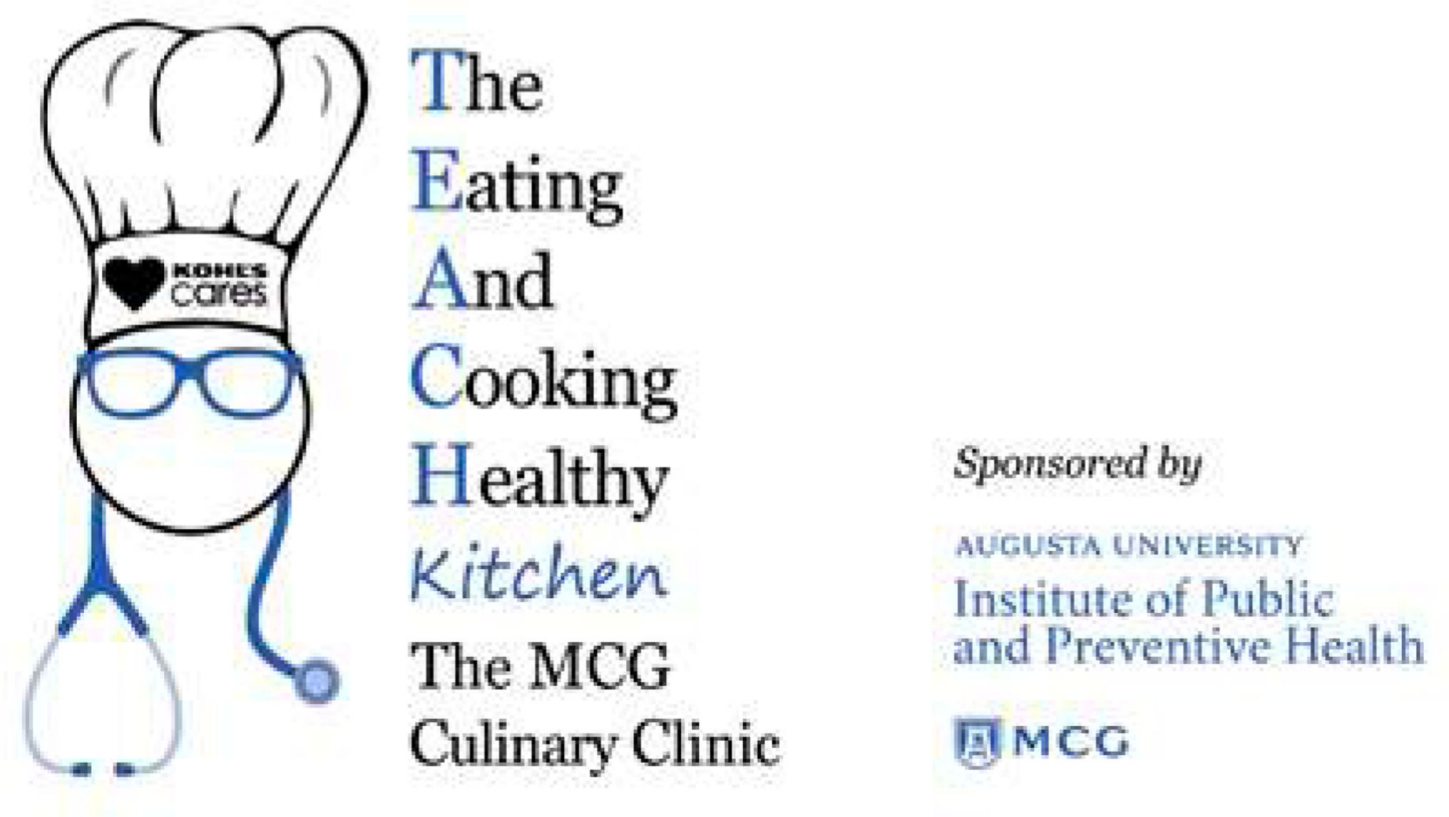
TEACH logo

**Figure 7:**
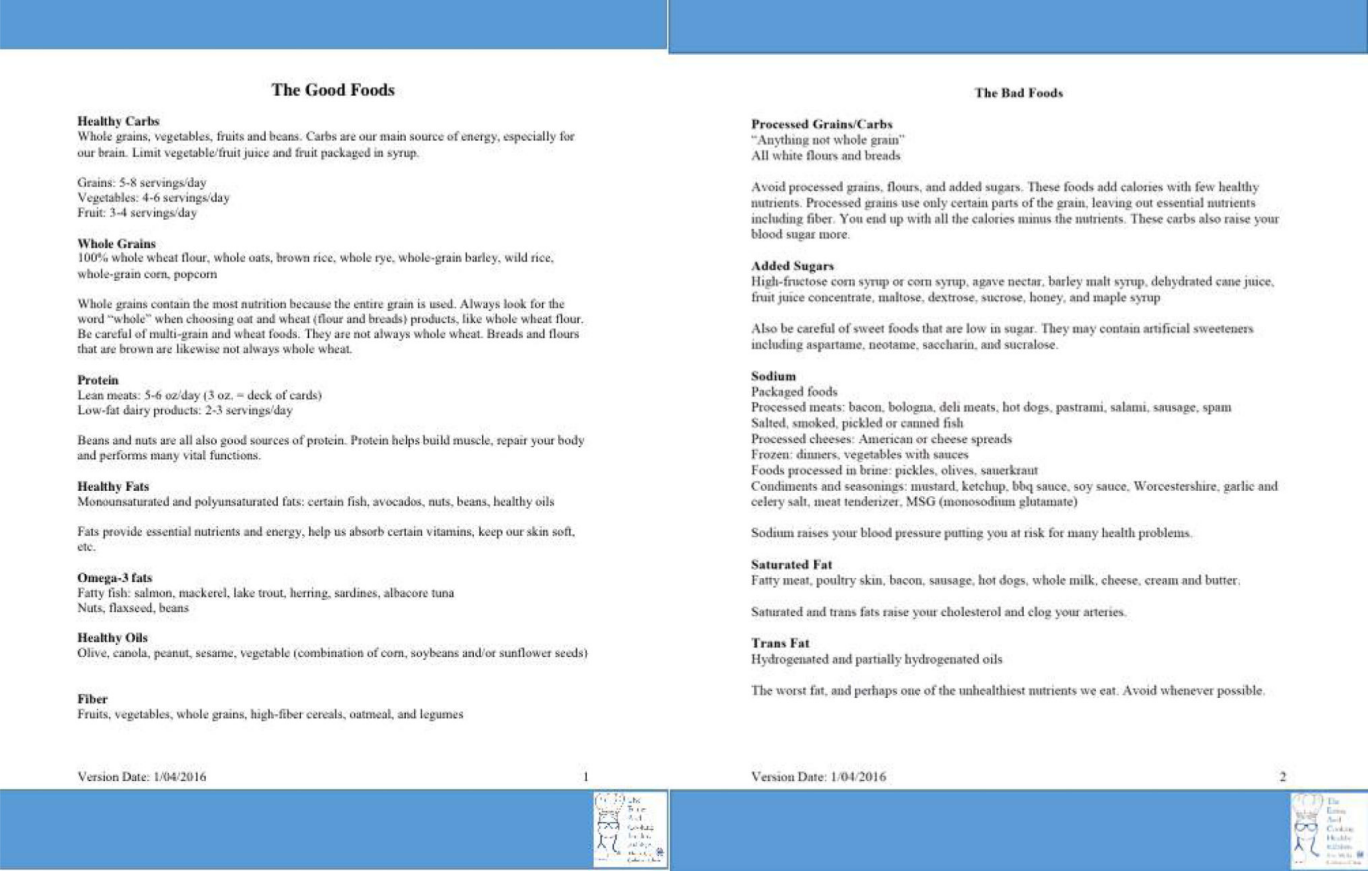
“The Good Foods vs The Bad Foods” nutrition educational handout

During the fall semester, the TEACH logo was designed with the Augusta University Division of Communications and Marketing (Figure 6), and nutrition educational materials with the official logo were created. Figure 7 is a handout that reviews healthy versus unhealthy foods, providing examples in each category as well as recommended servings.

TEACH coordinators researched healthy diet guidelines, and collaborated with nutrition experts to create educational materials. Healthy eating tips were taken from the Dietary Guidelines for Americans, which is published every 5 years by the US Department of Health and Human Services and the US Department of Agriculture (USDA). These guidelines reflect the most current state of nutrition science. Healthy diet guidelines published by the World Health Organization were reviewed as well.

TEACH coordinators also worked with Dr. Ansa to develop a research protocol for submission to the IRB at Augusta University. The proposed study gathers data with baseline and post-intervention surveys (see Appendix for full questionnaire) that assess differences in participants’ pre-and post-intervention knowledge, attitudes, and beliefs related to healthy eating. A protocol for TEACH Kitchen was submitted to the Institutional Review Board in December 2015 and was published in the fall of 2016 (White et al., 2016) after being approved in November 2016.

Currently, the TEACH study is underway. The cooking sessions on hypertension began in January 2017. Nine patients completed the four cooking sessions on this module. The next module begins in April 2017.

**Figure 8:**
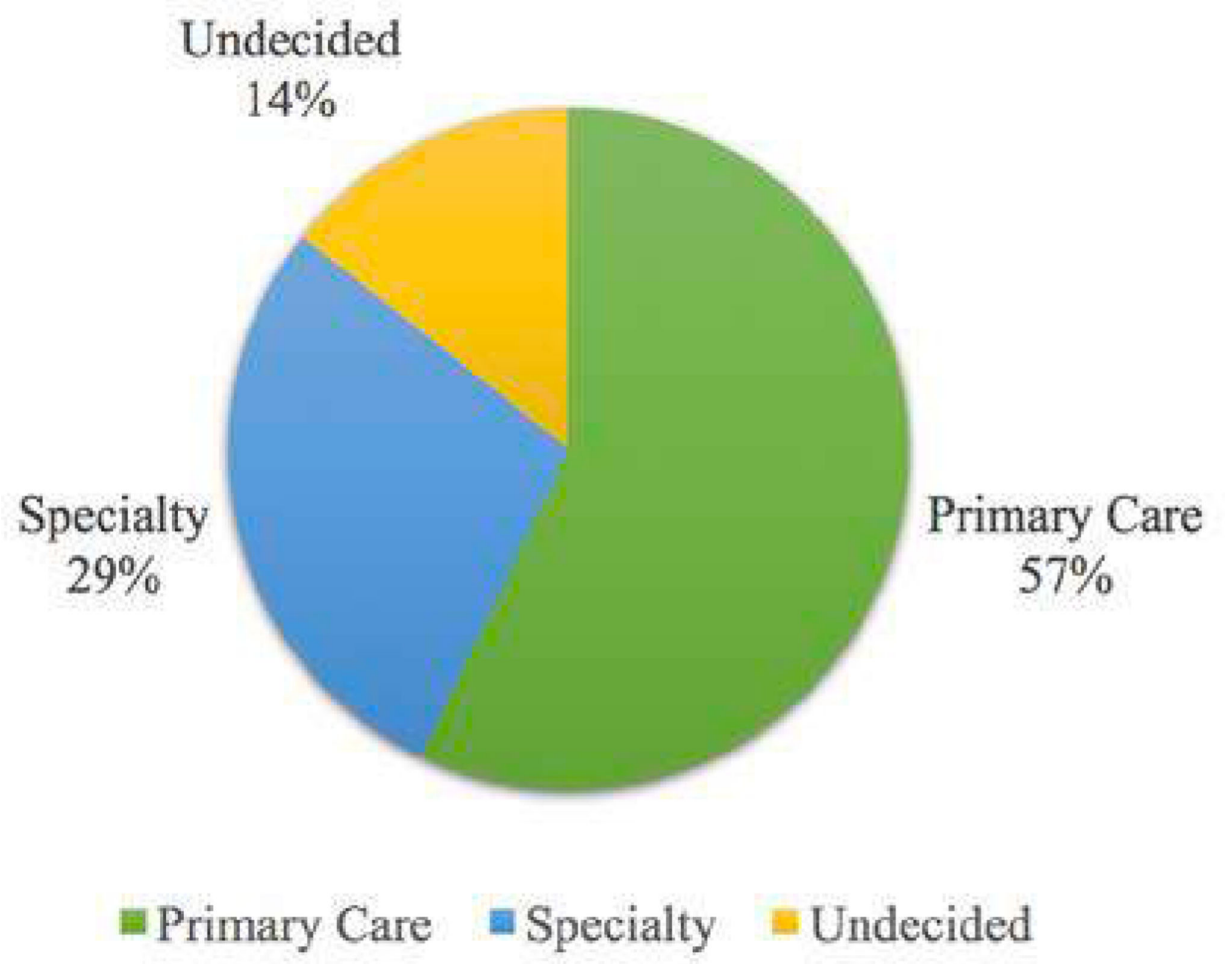
Specialty choice for first cohort of TEACH coordinators

At present, the first cohort of TEACH coordinators have been matched into residency programs. This provides an insight into what kind of students are attracted to being involved in medical school-based teaching kitchens. Among 7 students, 4 are entering a primary care field (Internal Medicine), 2 have chosen a specialty field, and 1 is undecided. Thus, more than half of the first cohort of TEACH coordinators are entering a primary care field in which physicians face the challenges of effective nutrition counseling (Figure 8).

Interestingly, a survey of the second and third cohort of TEACH coordinators revealed that a majority (75%) are interested in a specialty field.

**Figure 9:**
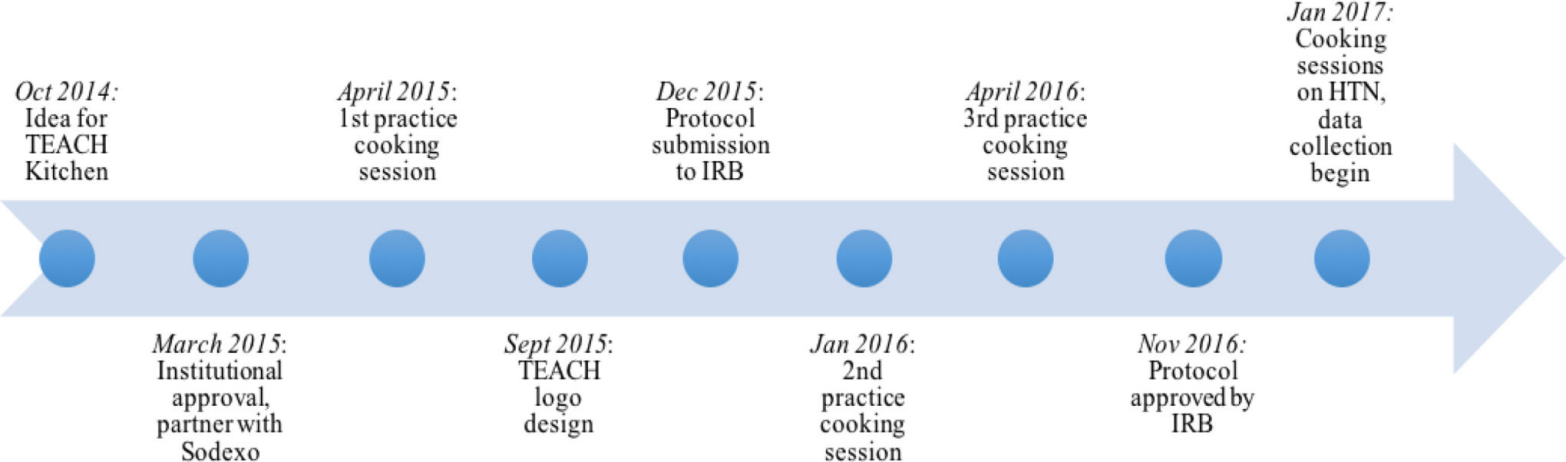
TEACH Kitchen milestones

In summary, TEACH Kitchen has grown from an idea to a full-fledged, IRB-approved study to examine how nutrition interventions may lead to healthier lifestyles and improved health outcomes. Figure 9 summarizes milestones in the timeline of TEACH Kitchen.

## RESULTS

**Figure 10:**
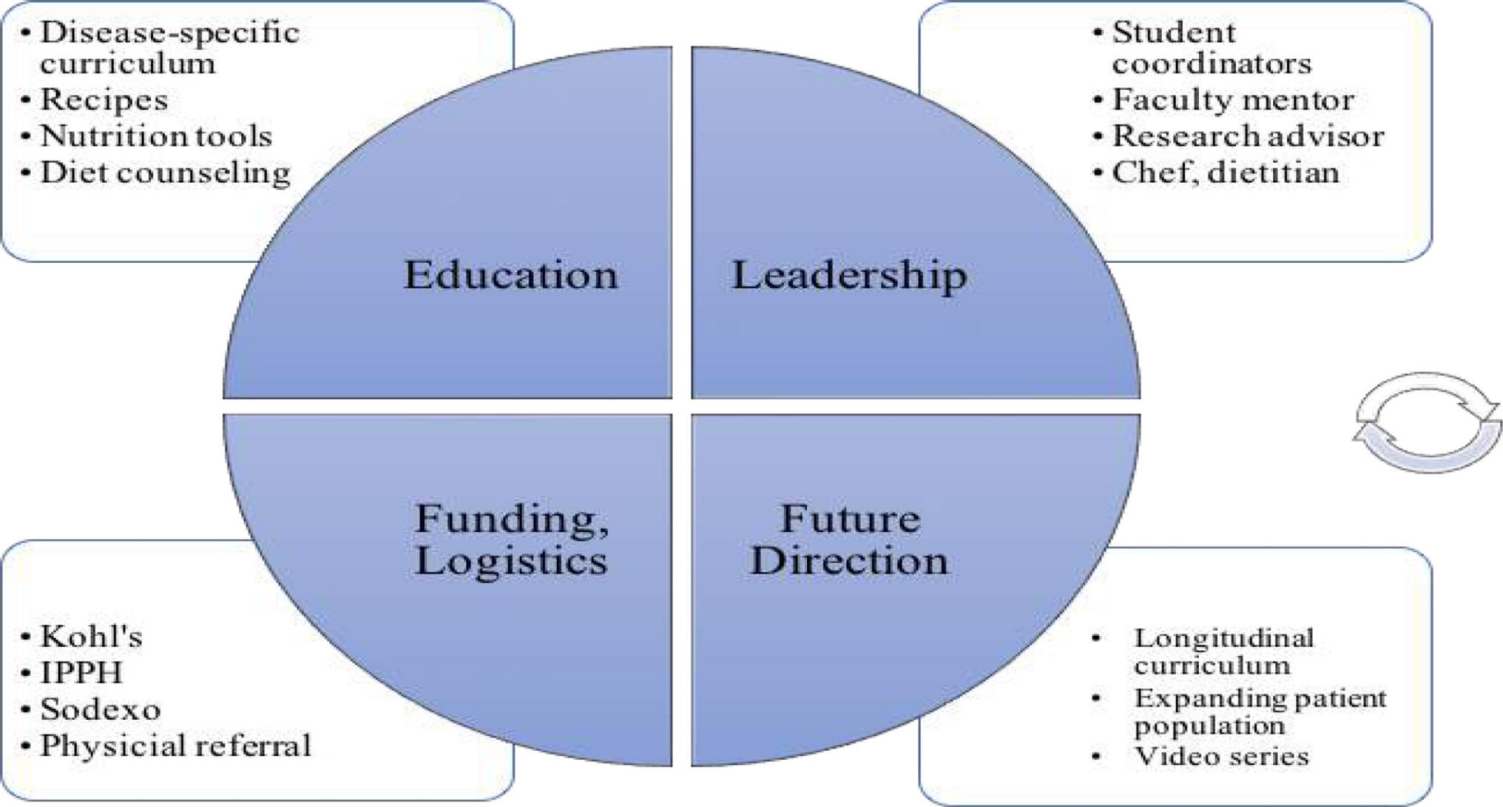
Focus areas of focus for TEACH Kitchen

The anticipated results of TEACH Kitchen are promotion of healthy eating in the Augusta community, prevention of obesity-related complications of chronic disease, and decreased prevalence of obesity for adolescents in the community. To date, 14 patients and 6 children have enrolled in TEACH Kitchen cooking classes. Approximately 99% of patients complete the entire module. Two areas of potential improvement are recruitment and retention of enrolled patients. Some rationales for why it is difficult to recruit patients include persisting inability to impress on patients the importance of healthy nutrition and cooking, lack of transportation, and lack of awareness of our program. We intend to address these issues by strengthening our partnerships with physicians and student-led clinics in the community, increased advertising, and continuing to grow our reputation as a patient-centered and patient-friendly healthy nutrition and cooking class in the Augusta area. Rationales for why it is difficult to retain patients include lack of transportation, loss of interest in the curriculum, or patient non-compliance for other socioeconomic factors. We hope to address these issues by recruiting more volunteers to lower the student-teacher ratio, so that patients feel like they have a more one-on-one learning experience. Transportation issues could potentially be addressed by setting up a TEACH Kitchen bus schedule through the university in the future. Our efforts to grow TEACH Kitchen over the past few years have involved several components. First, we planned and created the education component of the program. Education materials included a disease-specific curriculum (related to obesity, hypertension, hyperlipidemia, and diabetes mellitus), nutrition handouts, lectures, and healthy recipes. Second, we coordinated our efforts with the faculty, university, and other healthcare and food professionals. We also established relationships with various institutions and companies for funding, which is essential to our mission. In summary, there are four components that make TEACH Kitchen possible: education, leadership, funding and logistics, as well as plans for future direction. Figure 10 highlights these four areas of focus.

## DISCUSSION

Our goals are to continue building the program and enhancing its reputation in the Augusta community. We intend to involve students from other healthcare professions such as physician assistants, nursing, and dentistry. Involving other healthcare schools at Augusta University would also expand our potential pool of patients. Since MCG has campus locations outside of Augusta (Savannah, GA; Albany, GA; and Rome, GA), TEACH Kitchen may be able to expand to these satellite sites. Coordinators have communicated an interest of incorporating TEACH into the medical student curriculum. To reach patients who are unable to attend TEACH cooking sessions due to transportation issues, we are planning to develop an online video to be available to the public.

To determine the direction of future research, we plan to assess process, outcome, and impact evaluations. Evaluation of the process focuses on procedures implemented during TEACH, such as patient referrals, marketing to the student body and university, recruitment of study coordinators and volunteers, and funding. Process evaluation data are collected with pre- and post-intervention questionnaires related to student attitudes and beliefs.

Outcome evaluation provides data on the effectiveness of TEACH Kitchen. These data measure pre- and post-intervention changes in patients’ attitudes, clinical history, and dietary habits. Clinical variables such as BMI, HbA1c, blood pressure, and total cholesterol are compared (White et al., 2016). Impact evaluation assesses the long-term effect of participating in the TEACH program, and long-term impact is measured by repeating the outcome evaluation at three months post-intervention. These data will allow us to determine if the intervention leads to lasting lifestyle changes, rather than to transient modifications in behavior.

## IMPLICATIONS FOR PUBLIC HEALTH

TEACH Kitchen, a promising addition to the Augusta community, presents a method of combating the high prevalence of obesity and obesity-related chronic diseases in Georgia. Medical schools do not provide substantial education on nutrition, and the time constraints faced by practicing physicians may limit nutritional counseling, especially if physician attitudes and competence about nutrition education are low. Medical school-based cooking kitchens present a solution to this problem. Programs like TEACH Kitchen allow medical students to become familiarized with the community and acquaint them with giving nutrition advice. By practicing these behaviors, students are more likely to counsel patients more effectively in the future. Participation in the cooking sessions also raises awareness of special circumstances faced by those in the community. TEACH Kitchen can have a meaningful impact on improving public health by providing opportunities for patients to develop nutrition proficiency, and for future physicians to improve competence in nutrition counseling.

## APPENDIX

Questionnaire used in TEACH study to assess pre- and post-intervention knowledge, attitudes and beliefs about nutrition, as well as healthy eating and cooking.
